# Instruments for assessing healthcare professionals’ knowledge, attitudes and practices regarding deprescribing: a scoping review

**DOI:** 10.1007/s11096-026-02215-y

**Published:** 2026-08-21

**Authors:** Ana Carolina Figueiredo Modesto, Luis Phillipe Nagem Lopes, Mariana Del Grossi Moura, Alan Maicon de Oliveira, José Salvador Carrillo, Beatriz Birelli do Nascimento, Flávia Casale Abe, Luciane Cruz Lopes

**Affiliations:** 1 Postgraduate Program in Pharmaceutical Sciences - University of Sorocaba, Sorocaba, Brazil; 2 Hospital Clinics of the Federal University of Goiás, Goiânia, Brazil; 3 Pharmacy School, Federal University of Rio de Janeiro, Rio de Janeiro, Brazil; 4 Department of Human Medicine, San Juan Bautista Private University, Lima, Peru

**Keywords:** Attitudes and practices, Deprescriptions, Health knowledge, Health personnel, Surveys and questionnaires

## Abstract

**Introduction:**

Deprescribing is a multiprofessional process which aims to optimize medication use and improve patient safety, in which healthcare professionals' knowledge, attitudes, and practices (KAP) play a central role. The instruments used to assess KAP among nurses, pharmacists and physicians remain poorly characterised in terms of scope and measurement quality.

**Aim:**

To map and characterize the instruments used to assess healthcare professionals’ knowledge, attitudes, and practices regarding deprescribing.

**Method:**

A scoping review was conducted following Joaassess healthcaree (JBI) methodology and reported according to the PRISMA Extension for Scoping Reviews (PRISMA-ScR). Primary studies were eligible if they involved nurses, pharmacists or physicians and applied any instrument assessing KAP related to deprescribing, individually or in combination, regardless of setting or country. Nine electronic databases were searched, including two grey literature sources were searched from inception to September 2024, without restrictions on language or publication date using terms related to nurses, pharmacists, physicians, deprescribing, surveys and questionnaires, and health knowledge, attitudes, and practice Study selection and data charting were performed independently by three pairs of reviewers using a standardised extraction form capturing study characteristics, instrument details, KAP domains assessed, and reported measurement properties. Data was synthesised using descriptive statistics and narrative analysis. The review protocol was registered on the Open Science Framework.

**Results:**

Of the 3,821 records screened, 49 met the inclusion criteria. Over two-thirds of the included studies were published since 2020 (n = 33, 67.3%). The evidence base was concentrated in high-income countries (n = 36/46, 78.0%; three multi-country studies were not classified by income level), particularly in the Europe and Central Asia region (n = 19, 41.0%), and most often focused solely on physicians, while studies simultaneously involving all three professional groups (physicians, pharmacists, and nurses) were less frequent (n = 8, 16.0%). Instruments commonly assessed multiple KAP domains, especially knowledge, attitudes, and practices combined (n = 12, 30.0%), whereas evidence on psychometric properties was reported in only a minority of studies (n = 13, 26.5%), among these, internal consistency and content validity were the pair most frequently reported together (n = 4, 31.0%).

**Conclusion:**

Instruments assessing healthcare professionals’ KAP regarding deprescribing remain limited in scope and psychometric robustness. Expanding interprofessional coverage and strengthening validation efforts are key priorities for advancing research in this field.

**Supplementary Information:**

The online version contains supplementary material available at https://doi.org/10.1007/s11096-026-02215-y.

## Impact statements

- Most existing instruments assessing knowledge, attitudes and practices regarding deprescribing were developed and validated predominantly among physicians and in high-income countries, which may limit their applicability to pharmacists, nurses and professionals working in more diverse clinical and geographic settings.
- Psychometric evidence supporting these instruments remains limited, with most reporting only internal consistency and content validity; clinicians and researchers selecting a tool should weigh this gap against their intended use.
- Using valid and contextually appropriate instruments to measure knowledge, attitudes and practices may support safer, more coordinated and patient-centred deprescribing practices.

## Introduction

Deprescribing is broadly defined as the planned process of withdrawing an inappropriate medication, supervised by a healthcare professional, with the goal of managing polypharmacy and improving outcomes, and may also involve dose reduction or tapering depending on the clinical context [1]. Nearly half of older adults are exposed to polypharmacy, which has been linked to adverse outcomes such as falls, hospitalizations, and higher healthcare costs [2]. In this context, deprescribing has emerged as an important strategy to optimize medication use and improve patient care [2, 3]. Deprescribing is a structured and patient-centred process that requires careful assessment of benefits and harms, shared decision-making, and continuous monitoring [4, 5]. Consequently, the successful implementation of deprescribing depends on factors related to the clinical context, the organization of health systems, and the practices of healthcare professionals [4, 5].

Deprescribing involves clinical judgments that go beyond simply identifying potentially inappropriate medications [4, 6]. It requires recognizing which patients are most likely to benefit [4], prioritizing medications within complex therapeutic regimens, and negotiating decisions with patients and caregivers [7]. Evidence suggests that barriers to deprescribing occur at multiple levels, including limited time, fragmented clinical information, fear of adverse consequences, concern about peer judgment, and perceived resistance from patients [3, 8]. Many of these barriers are closely related to how healthcare professionals manage uncertainty and approach medication discontinuation in routine practice [9]. Understanding what they know, how they view deprescribing, and how these perceptions translate into action is essential for understanding its implementation.

A commonly used model in health sciences to explore behavioral determinants is the Knowledge, Attitudes, and Practices (KAP) framework [10]. This framework conceptualizes professional behavior as the interaction between what individuals know, how they perceive and value a given practice, and how they act in routine care [10, 11]. Rather than viewing behavior as a single outcome, the KAP framework helps clarify how knowledge gaps, attitudinal resistance, or contextual pressures may shape implementation processes in different ways [11]. Because KAP instruments are often used to identify modifiable determinants of professional behaviour, understanding their scope and measurement properties is essential for determining whether they can inform deprescribing implementation strategies across different healthcare contexts [10].

The KAP framework has been applied across a wide range of health topics, including infection prevention [12], vaccination uptake [13], antimicrobial resistance [14], and polypharmacy management [15], where understanding professionals’ knowledge, perceptions, and routine practices is essential for informing educational and implementation strategies. In the context of deprescribing, the KAP framework is particularly relevant, as medication discontinuation requires not only clinical competence but also confidence, risk appraisal, and willingness to reconsider established prescribing patterns [4, 5]. Although this framework has been used to deprescribing research, most studies have focused on patients and caregivers, exploring their perceptions and experiences regarding medication discontinuation [16–18]. When healthcare professionals are included, the literature has predominantly focused on physicians [19–21], whereas studies involving pharmacists and nurses remain scarce [22]. Pharmacists and nurses play a key role in the medication use, which is inherently complex, supporting pharmacotherapy review and monitoring for potential adverse events throughout the deprescribing process [22]. Whether existing instruments adequately capture the conceptual scope of the KAP framework, professional coverage, and measurement is still uncertain.

## Aim

To address this gap and provide a comprehensive overview of existing assessment approaches, this scoping review aimed to map and characterize the instruments used to assess healthcare professionals’ knowledge, attitudes, and practices regarding deprescribing.

## Method

### Study design

This scoping review adhered to the Joanna Briggs Institute (JBI) methodology [23, 24] and was reported in line with the PRISMA Extension for Scoping Reviews (PRISMA-ScR)[25]. The protocol was registered a priori on the Open Science Framework platform [26] and has also been published in a peer-reviewed journal [27].

### Review question

The review question was: What instruments are available to assess healthcare professionals’ knowledge, attitudes, and practices regarding deprescribing? The population comprised healthcare professionals involved in medication-related care, including physicians, pharmacists, and nurses. The concept was instruments assessing knowledge, attitudes, and/or practices related to deprescribing. The context included any healthcare setting, including primary, secondary, and tertiary care.

### Eligibility criteria

The eligibility criteria were defined a priori and are publicly available in the published protocol[26, 27]. Regarding concept, this review considered any definition of deprescribing, as well as studies addressing any component of the deprescribing process, individually or in combination. Any research instrument or questionnaire, validated or not, was considered an eligible tool; tools designed to assist the deprescribing process itself (e.g., STOPP-START) were excluded. Eligible instruments had to assess at least one of the following domains: knowledge, attitudes and/or practices, individually or in combination [27]. A summary of the inclusion and exclusion criteria applied in this review is presented in Supplementary Material 1.

### Search strategy

A comprehensive search was conducted in the following electronic databases: PubMed, Virtual Health Library, Cochrane Library, Scopus, Web of Science, Embase, and CINAHL. To capture relevant grey literature, searches were also performed in Google Scholar and ProQuest.

The search strategy was initially developed for PubMed using a combination of MeSH terms and synonyms related to the population, concept, and context of the review. Boolean operators (AND, OR) were applied to combine the search terms appropriately. Each concept was decomposed into its corresponding MeSH heading, entry terms, and related terms, which were validated against three sentinel articles, that is, studies the strategy was expected to retrieve; all three were successfully retrieved using the resulting terms. The strategy was then reviewed by a specialized librarian and adapted for use across all other databases according to each database's own controlled vocabulary. The full search strategies for each database are available in Supplementary Material 2.

A supplementary manual search was carried out by screening the reference lists of all included studies, as well as of relevant systematic reviews. The searches were conducted in September 2024 and included all records available from the inception of each database, without restrictions on language or publication date.

### Study selection

All citations retrieved from the database searches were first imported into Zotero for deduplication and then uploaded into Rayyan software for screening. Of the 7,655 records retrieved across the nine databases searched, 3,834 duplicates were identified and removed in Zotero, yielding 3,821 unique records that were uploaded into Rayyan for title and abstract screening. Title and abstract screening, as well as full-text assessment, were performed independently by three pairs of reviewers. An additional seven duplicate records, corresponding to the same study published in more than one language, were identified during full-text assessment, as this type of duplication could not be detected by automated deduplication. Each phase of the selection process was preceded by a calibration exercise to ensure consistency among reviewers. Disagreements at any stage were resolved by consensus or with the involvement of a third reviewer when needed. Reasons for exclusion at the full-text review stage were documented.

### Data extraction

Data were extracted using a standardized form designed to capture information on study characteristics, population characteristics, and the instruments assessed (Supplementary Material 3). Extraction was performed by three pairs of reviewers working independently. Each item was classified as knowledge (understanding of deprescribing), attitude (beliefs and predispositions toward deprescribing, regardless of what professionals know or do), or practice (actions actually implemented in clinical care), following a framework previously developed [28], and items fitting none of these domains were classified as 'other'. Prior to the formal extraction phase, a calibration exercise was conducted with five articles per reviewer to test the form, align interpretations, and ensure consistency in applying the criteria. Any discrepancies identified during calibration or throughout the extraction process were resolved by consensus; when consensus could not be reached, an experienced third reviewer acted as an arbitrator.

### Data analysis

Extracted data were synthesized using descriptive analysis. Study characteristics, including publication year, country and income classification, geographic region, study setting, professional categories and study design were summarized using absolute and relative frequencies. Instruments were analyzed according to the domains assessed, of KAP and whether these domains were evaluated individually or in combination. Measurement properties were categorized using terminology consistent with established guidance [29] for health measurement instruments, including internal consistency, reliability, content validity, criterion validity, and construct validity when reported. For descriptive purposes, a three-level validation classification was applied based on how many of these five properties were reported. Level 1 = only one property assessed; Level 2 = two or more properties assessed, without construct validity; Level 3 = two or more properties assessed, including construct validity. This three-level classification was developed by the review team based on The Consensus-based Standards for the Selection of Health Measurement Instruments (COSMIN) principles and is intended to provide a descriptive overview of the depth of psychometric evidence available for each instrument, not to establish definitive quality judgements. Critical appraisal of the included studies was not performed, as this is not a requirement of scoping review guidelines. The aim of this review was to map the instruments used and their reported measurement properties, rather than to assess the effectiveness or quality of interventions, consistent with JBI methodology. Analyses were conducted using R software (version 4.5.1).

## Results

**Fig. 1:**
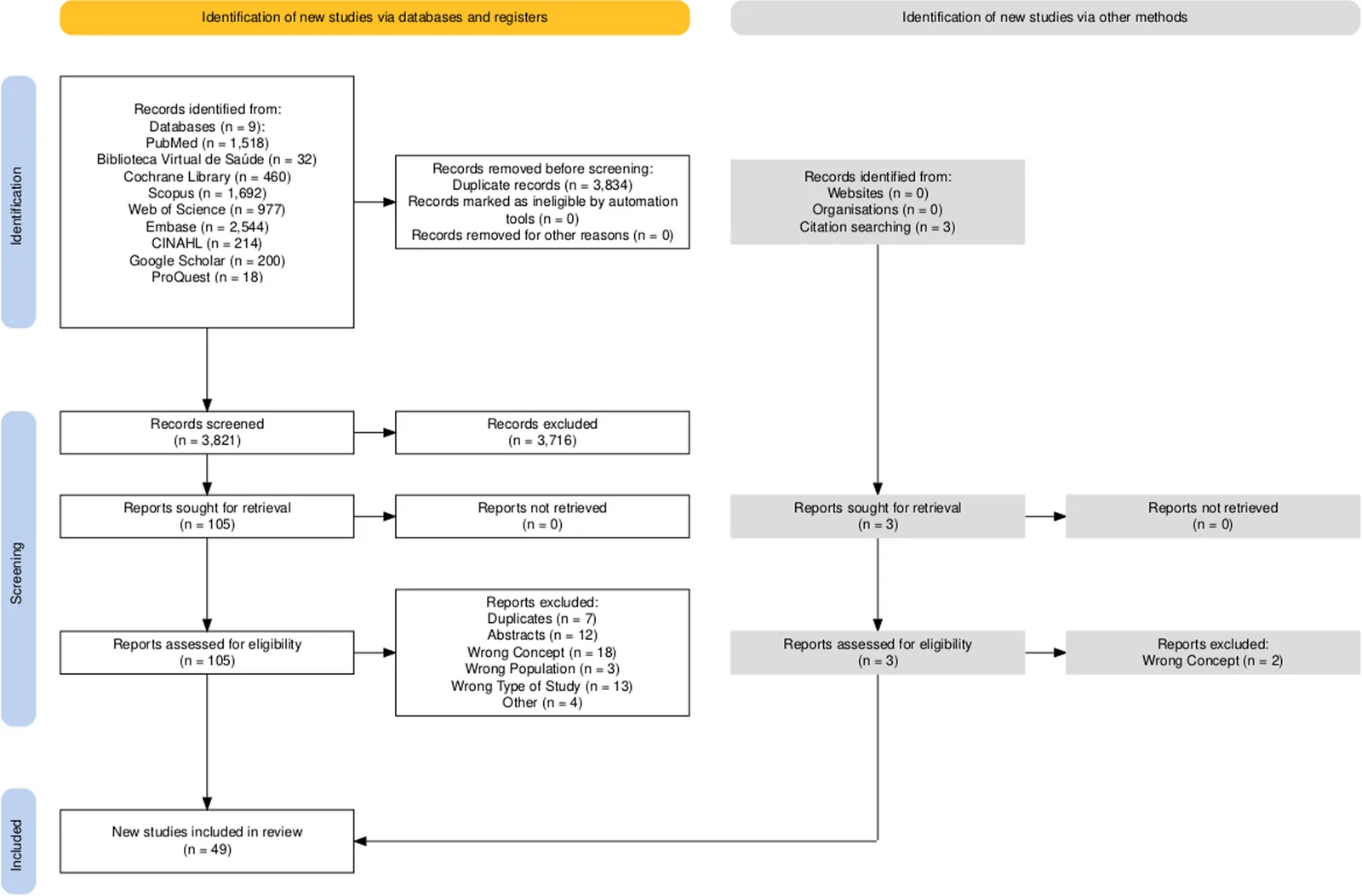
PRISMA flowchart

The searches were conducted across nine databases and identified 7,655 records. After removing 3,834 duplicates, 3,821 records underwent title and abstract screening, of which 105 were retrieved for full-text assessment alongside three records identified through other sources. Following full-text review, 59 records were excluded, most frequently due to non-compliance with the concept criterion (n = 18), ineligible study design (n = 13), and availability solely as conference abstracts (n = 12). An additional seven duplicate records, corresponding to the same study published in more than one language, were identified at this stage, as this type of duplication could not be detected by automated deduplication. A total of 49 studies met all inclusion criteria and were included in this scoping review. The complete study selection process is illustrated in Fig. 1.

### Study characteristics

A total of 49 studies met the eligibility criteria and were included in this scoping review. The included studies were published between 1996 and 2024, with the majority (67.3%, n = 33) published since 2020 [30–62]. The included studies were conducted across 22 different countries, with a predominance of studies from the United States [40, 42, 47, 48, 57, 60, 63] (n = 7, 14.0%) and India [19, 36, 38, 64] (n = 4, 8.2%). Three studies [56, 58, 65] (6.1%) were conducted across multiple countries. Regarding country income classification, most studies [33–35, 39–42, 44–55, 57, 59–61, 63, 66–77] (n = 36, 78.0%) were conducted in high-income countries. In terms of geographic distribution, the majority of studies were conducted in Europe and Central Asia [33–35, 39, 41, 44–46, 49–51, 55, 59, 61, 71, 72, 74, 75, 77] (n = 19, 41.0%) and North America [40, 42, 47, 48, 57, 60, 63, 69, 73, 76] (n = 10, 22.0%).

In terms of study setting, the most frequent location was primary care [33, 39–42, 44, 47, 51, 55, 60, 63, 66, 68, 70–72, 74, 75] (n = 18, 37.0%), followed by hospitals [30, 32, 36, 38, 43, 67] (n = 6, 12.0%) and community pharmacies (n = 3, 6.0%). Seven studies [19, 50, 56, 62, 64, 65, 77] (14.0%) did not report the setting in which data collection took place.

With respect to study design, the majority of studies were cross-sectional [30–33, 35–46, 48–53, 55–72, 74–77] (n = 44, 90.0%). Three studies (6.1%) were methodological, aimed at cross-sectional validation of an instrument [19, 34, 54], and two (4.1%) employed a mixed-methods design [47, 73].

### Population characteristics

Physicians were the most frequently studied professional category, with most studies focusing solely on this group [19, 33–38, 41–44, 46, 50, 56–58, 60, 62, 65, 67–75, 77] (n = 32, 65.0%), whereas studies exclusively involving pharmacists were less frequent [49, 52, 53](n = 3, 6.1%). Only eight studies [30–32, 40, 54, 63, 66, 76] (16.0%) simultaneously assessed all three professional groups, physicians, pharmacists, and nurses. Regarding the number of eligible healthcare professionals (nurses, pharmacists, and physicians), the studies showed a range of 30–1,415, with a mean of 297 (SD = 326).

### Instrument characteristics

Among the 49 included studies, 40 (82.0%) [19, 31–34, 36–51, 53–61, 64–68, 70, 73–76] reported the instrument in sufficient detail, either within the main text or supplementary materials, to allow full identification of all questionnaire items. Among these 40 studies which reported sufficient instrument detail, approximately one third (n = 12; 30.0%) simultaneously evaluated KAP [34, 36–39, 43, 45, 47, 49, 58, 59, 61, 67, 70]. When domains were assessed in isolation, only attitudes [19, 32, 33, 44, 54, 64, 65, 68, 74, 76] (n = 10; 25.0%) and practices [50, 51, 73](n = 3; 7.5%) were evaluated separately, whereas knowledge was never assessed as a standalone domain in any of the instruments used in these 40 studies. The remaining domain combinations observed among the included instruments are presented in Supplementary Material 4.

Regarding validity evidence, only about one third of the included studies (n = 13; 26.6%) reported any form of validity assessment for the instruments used [19, 31, 34, 37, 43, 47, 51, 52, 54, 60, 61, 68, 76]. Among these studies which reported any form of validity assessment, internal consistency and content validity were the pair most frequently reported together, occurring in 4 studies [31, 37, 43] (n = 4, 31.0%). Internal consistency[51, 76] and content validity [60, 68] were the most frequently reported metrics when assessed individually, each utilized in two studies (n = 2, 15.0% each). A small proportion of studies exhibited four psychometric measures. These most complex analyses were observed in one study [47] which assessed internal consistency, content validity, criterion validity, and construct validity (n = 1, 7.7%) (Table 1).

**Table 1:** Psychometric properties and validation level of instruments identified in included studies

Study	Instrument	Number of Questions	Internal Consistency	Reliability	Content Validity	Construct Validity	Criterion Validity	Validation Level
Saka et al., 2024 [31]	NR	38	✔	-	✔	-	-	2
Agosti et al., 2024 [34]	Medical Attitudes Towards Deprescribing Questionnaire (MATD-Q)	43	✔	✔	-	✔	-	3
White et al., 2019 [68]	NR	10	-	-	✔	-	-	1
Abu-Fahra et al., 2024 [37]	NR	38	✔	-	✔	-	-	2
Akande-Sholabi et al., 2023 [43]	NR	48	✔	-	✔	-	-	2
Niznik et al., 2022 [47]	NR	35	✔	-	✔	✔	✔	3
Lopes et al., 2021 [51]	NR	16	✔	-	-	-	-	1
El-Dahiyat et al., 2023 [52]	NR	32	✔	-	✔	-	-	2
Shresta et al., 2022 [54]	Health Care Professionals’ Attitudes Towards Deprescribing (HATD)	23	✔	-	✔	✔	-	3
Farrell et al., 2018 [76]	NR	10	✔	-	-	-	-	1
Bhat et al., 2018 [19]	Perceptions, Attitudes and Challenges of Physicians towards Deprescribing (PACPD-12)	12	-	✔	✔	-	-	2
Norton et al., 2023 [60]	Understanding Physicians Deprescribing Decisions	14	-	-	✔	-	-	1
Buzancic et al., 2022 [61]	Comprehensive healthcare provider’s opinions, preferences, and attitudes towards deprescribing questionnaire (CHOPPED)	44	✔	-	✔	✔	-	3

✔:Assessed and reported

-:Not assessed or not reported

Instruments without a formal name are indicated as not reported (NR)

Internal consistency: degree of interrelatedness among an instrument's items [28]

Reliability: proportion of variance in scores attributable to genuine differences between respondents (e.g., test–retest or inter-rater stability)[28]

Content validity: extent to which an instrument's content adequately reflects the construct it intends to measure[28]

Construct validity: extent to which an instrument's scores relate to other variables as theoretically expected for the construct being measured[28]

Criterion validity: extent to which an instrument's scores correspond to an external, independently verified measure of the same construct[28]

Among the 13 instruments with reported psychometric evidence, internal consistency and content validity were the properties most frequently assessed (10/13 each). In contrast, reliability and criterion validity were the properties least frequently reported, assessed in only 2/13 and 1/13 instruments, respectively. The instrument reported by Niznik et al. [47] showed the most comprehensive validation profile, being the only one for which criterion validity was also assessed, alongside internal consistency, content validity, and construct validity. The remaining Level 3 instruments, Agosti et al. [34], Shresta et al. [54], and Buzancic et al. [61], each reported three properties but did not include criterion validity, indicating that even the most extensively validated instruments in this review lack evidence on this specific property.

A comprehensive summary of the characteristics and main findings of studies reporting KAP assessment instruments is provided in Supplementary Material 5.

## Discussion

### Main findings

This review shows a mismatch between the growing use of KAP instruments in deprescribing research and the limited reporting of their conceptual scope and measurement properties. Structured questionnaires with predefined items predominated among the included studies, suggesting that most studies relied on standardized self-report measures to assess these constructs. However, only a limited number of studies reported any form of psychometric evaluation, which indicates that the measurement properties of many instruments remain poorly documented. Overall, the literature reflects a field in which instruments are widely used, but the systematic reporting of validity evidence remains inconsistent.

### Study characteristics

Regarding publication year, although the review covers almost three decades, the evidence is concentrated in the most recent years, particularly after 2020. These findings may be partly explained by population ageing and the increasing prevalence of multimorbidity [78], which have intensified the complexity of clinical care and medication management [4]. In this context, studies examining healthcare professionals’ knowledge, attitudes, and practices have gained relevance as a way to support the implementation of deprescribing in complex clinical settings. These temporal trends are consistent with literature, which document a growth in deprescribing-related publications over the past decade [79]. These findings suggest that deprescribing has emerged as a consolidated and expanding field of research.

The included studies were mostly conducted in high-income countries, for example Europe and North America, in line with results of a bibliometric analysis, with research concentrated in the United States, Australia, and Canada [80]. This distribution may reflect differences in health systems capacity to support medication management, as deprescribing requires coordinated collaboration among professionals and reliable access to comprehensive medication and clinical records [81]. Evidence from low-income countries suggests that electronic health record systems are still at early stages, often limited by infrastructure, interoperability, and integration challenges [82]. In such contexts, deprescribing may be less systematically integrated into routine care, limiting both its implementation and the development of related assessment instruments. This also raises concerns about the transferability of instruments developed in high-income settings which may not fully reflect differences in professional roles, regulatory frameworks, access to clinical records, and care organization in other health systems [82].

Primary care was the predominant setting in which instruments assessing healthcare professionals’ knowledge, attitudes, and practices regarding deprescribing were applied. A recent review on deprescribing in older adults reported a similar pattern, with most studies conducted in primary care settings [2]. This predominance suggests that deprescribing has been primarily framed as a longitudinal process integrated into ongoing medication stewardship and care coordination [4, 81]. In primary care, periodic reassessment of complex therapeutic regimens, particularly among patients with multimorbidity, is a central component of clinical practice, which may help explain the higher number of studies conducted in this setting [7, 83]. Consequently, the available instruments tend to reflect the realities of primary care practice, while factors specific to hospital or transitional care settings remain less frequently represented.

### Population characteristics

Most studies applied KAP instruments primarily to physicians, with pharmacists and nurses included less frequently. Although deprescribing has been investigated in different professionais settings, limited discussion has focused on how KAP instruments are applied across professional groups. This pattern may reflect the physician-centred structure that still characterizes many health systems, where formal authority over prescribing and medication discontinuation remains largely concentrated within medical practice [84]. Although collaborative care models have expanded [83], decision-making hierarchies continue to position physicians as the principal actors responsible for pharmacotherapy management [84]. As a result, instruments designed primarily for physicians may not may fail to capture the practical contributions of other professionals, such as pharmacists' involvement in medication review and counselling, or nurses' role in monitoring patients after a medication is discontinued, reinforcing the need for instruments that reflect deprescribing as a shared, multiprofessional responsibility.

### Instrument characteristics

Regarding the domains assessed, approximately one third of the included studies simultaneously evaluated knowledge, attitudes, and practices. Although the KAP framework is conceptually structured as an integrated model linking cognitive, attitudinal, and behavioral dimensions[10], its application in the included studies was frequently partial. Methodological guidance on KAP surveys suggests that these domains are theoretically interrelated, with knowledge informing attitudes and attitudes affecting practices [10, 11]. When domains are assessed separately, the explanatory coherence of the model may be weakened, limiting the ability to capture the full range of determinants underlying professional behavior[10, 11]. The fragmented application may also have implications for construct validity, as partial measurement risks under-representing the multidimensional nature of the KAP framework. Consequently, existing instruments may offer only a partial view of the factors influencing deprescribing implementation.

Regarding validity evidence, approximately one third of the included studies reported some form of psychometric evaluation, most commonly internal consistency and content validity, assessed either individually or in combination. Unlike diagnostic scales that can be evaluated against an established gold standard [29], many KAP surveys do not have an external criterion for direct comparison, since knowledge, attitudes, and practices are frequently context-specific and descriptive constructs. This may partly explain the predominance of content validity and internal consistency in the included studies. As most studies used cross-sectional designs with a single administration of the questionnaire, the emphasis on these properties is methodologically coherent, as they are commonly prioritized in early or exploratory phases of instrument development [84]. However, content validity in this early stage should be regarded as foundational rather than sufficient on its own, requiring clear construct definition, expert review, and, ideally, input from the target user group to be considered robust [83]. Because different measurement properties capture different aspects of instrument quality, internal consistency alone should not be taken as enough evidence of validity. Internal consistency reflects reliability, not whether the items represent the intended construct, and can remain high even when item content is too narrow or repetitive [29]. This can affect comparability across studies and also limit the use of these instruments for evaluating KAP in deprescribing context, especially when conceptual development and psychometric evidence are still limited.

### Strengths and limitations

This review has some limitations. The synthesis was based on information explicitly reported in the included studies, but some psychometric procedures may have been conducted but not clearly reported on studies. The heterogeneity of instruments, domains, response scales, and target professional groups limited the comparability of findings. Most studies were from high-income countries and focused on physicians, so the findings may not fully apply to other settings. Despite these limitations, this review has important strengths. It brings together the available instruments used to assess healthcare professionals’ knowledge, attitudes, and practices regarding deprescribing, while considering aspects such as conceptual scope, professional representation, geographic distribution, and reported psychometric evidence. The review was conducted and reported according to established methodological guidance for scoping reviews, which supports the transparency and reproducibility of the study. By examining how KAP domains have been operationalized and validated, this work offers a coherent overview of current measurement practices and identifies areas for methodological refinement.

### Implications for research and practice

These findings suggest that future studies should broaden the geographic and professional representation of KAP assessments in deprescribing. Most instruments have been developed and applied in high-income settings, and may not reflect the realities of health systems with different organizational structures, resources, and care pathways. Expanding research to include a wider range of professional groups and healthcare contexts may help ensure that KAP instruments better reflect the diversity of clinical practice. Future instruments would also benefit from being explicitly grounded in a theoretical framework and validated with methods appropriate to their stage of development, since most of the psychometric evidence identified here reflected early-stage properties rather than more robust validation. More detailed reporting of instrument domains, response formats and scoring would also support comparability across future studies in this field.

## Conclusion

This scoping review examined the instruments used to assess healthcare professionals’ knowledge, attitudes, and practices regarding deprescribing. Overall, the literature is concentrated in high-income countries, most studies focus on physicians, and the three KAP domains are not always assessed together. Although these instruments are widely used, information about their psychometric properties is reported inconsistently and is usually limited to basic validation measures. Future studies should prioritize the development, adaptation, and validation of theoretically grounded, interprofessional KAP instruments that can be applied across diverse healthcare systems and used to inform deprescribing implementation strategies.

## Supplementary Information

Below is the link to the electronic supplementary material.

Supplementary file1 (DOCX 322 kb)

Supplementary file2 (DOCX 141 kb)

Supplementary file3 (DOCX 148 kb)

Supplementary file4 (DOCX 216 kb)

Supplementary file5 (DOCX 4871 kb)

## Data Availability

No datasets were generated or analysed during the current study.
