# Supplementary material for: Instruments for assessing healthcare professionals’ knowledge, attitudes and practices regarding deprescribing: a scoping review
Source: Int J Clin Pharm. 2026 Aug 21;48(6):2201–11. doi: 10.1007/s11096-026-02215-y (PMC13731477; doi:10.1007/s11096-026-02215-y)
Supplement: Supplementary file 1 [file 11096_2026_2215_MOESM1_ESM.docx]

| **Table 1: Table 1: Eligibility criteria according to the Population, Concept and Context framework** | | |
| --- | --- | --- |
|  | **Inclusion** | **Exclusion** |
| Population | Studies conducted with healthcare professionals directly involved in medication process (nurses, pharmacists, physicians) | - Studies in which healthcare professionals cannot be identified. - Studies involving healthcare professionals, without specifying which type, will be included only if the results are presented separately for those who directly manage patient medication - Studies conducted with healthcare students |
| Concept | Studies with any definition of deprescribing, as well as studies which any component of the deprescribing process is present, either individually or in combination | - None |
|  | This review will consider as a tool, any research instrument or questionnaire, validated or not | - Studies with tools that assist in the deprescribing process, such as STOP-START |
|  | Knowledge (concepts, processes, tools, outcomes); Attitudes (barriers, facilitators); Practice (medication characteristics, patient context, environmental context, provider context) | - Studies that do not directly address the conceptual framework domains |
| Context | Any level of patient healthcare (primary, secondary, or tertiary) | - None |
| Study Type | Primary empirical research studies (randomised controlled trials, cohort studies, cross-sectional, mixed methods studies ) | - Qualitative studies - Systematic reviews or other types of reviews |
