# Supplementary material for: Instruments for assessing healthcare professionals’ knowledge, attitudes and practices regarding deprescribing: a scoping review
Source: Int J Clin Pharm. 2026 Aug 21;48(6):2201–11. doi: 10.1007/s11096-026-02215-y (PMC13731477; doi:10.1007/s11096-026-02215-y)
Supplement: Supplementary file 2 [file 11096_2026_2215_MOESM2_ESM.docx]

**Search Strategy**

| **Databasess** | **Search date** | **Search strategy** | **# of records retrieved** |
| --- | --- | --- | --- |
| Medline/PubMed | 12/09/2024 | ("nurses"[MeSH Terms] OR "nurse*"[Title/Abstract] OR "registered nurses"[Title/Abstract] OR "pharmacists"[MeSH Terms] OR "pharmacist*"[Title/Abstract] OR "clinical pharmacist*"[Title/Abstract] OR "community pharmacists"[Title/Abstract] OR "physicians"[MeSH Terms] OR "physician*"[Title/Abstract] OR "general practitioner"[Title/Abstract] OR "doctor*"[Title/Abstract]) AND ("deprescriptions"[MeSH Terms] OR "deprescriptions"[Title/Abstract] OR "deprescribing"[Title/Abstract] OR "inappropriate prescribing"[MeSH Terms] OR "inappropriate prescribing"[Title/Abstract] OR "De-prescribing"[Title/Abstract] OR (("medication*"[Title/Abstract] AND "discontinu*"[Title/Abstract]) OR "withdraw*"[Title/Abstract])) AND ("surveys and questionnaires"[MeSH Terms] OR "questionnaire*"[Title/Abstract] OR "survey*"[Title/Abstract]) AND ("health knowledge, attitudes, practice"[MeSH Terms] OR "Knowledge"[Title/Abstract] OR "attitude*"[Title/Abstract] OR "practice*"[Title/Abstract] OR "Attitude of Health Personnel"[MeSH Terms])  **Translations**  **Nurses[MeSH Terms]:** "nurses"[MeSH Terms]  **Pharmacists[MeSH Terms]:** "pharmacists"[MeSH Terms]  **Physicians[MeSH Terms]:** "physicians"[MeSH Terms]  **deprescriptions[MeSH Terms]:** "deprescriptions"[MeSH Terms] | 1518 |
| Biblioteca Virtual de Saúde | 13/09/2024 | (mh:(farmacêuticos)) OR (farmacêutico*) OR (farmacêutico* clínico*) OR (farmacêutico* comunitário*) OR (mh:(enfermeiras e enfermeiros)) OR (enfermeira*) OR (enfermeiro*) OR (mh:(médicos)) OR (médico*) AND (mh:(desprescrições)) OR (desprescrições ) OR (deprescrição) OR (deprescrições ) OR (desprescrição) AND (mh:(inquéritos e questionários)) OR (inquéritos e questionários) OR (enquetes e questionários) OR (inquérito*) OR (enquete*) OR (questionário*) AND (mh:(conhecimentos, atitudes e prática em saúde)) OR (conhecimentos, atitudes e prática em saúde) OR (atitudes e prática em saúde) OR (atitudes e práticas em saúde) OR (cap) OR (conhecimentos, atitudes e práticas em saúde) OR (pesquisas cap) OR (conhecimento*) OR (atitude*) OR (prática*) OR (mh:”Atitudes do pessoal de saúde”) | 32 |
| Cochrane Library | 13/09/2024 | ([mh nurses] OR nurse*:ti,ab OR "registered nurses":ti,ab OR [mh pharmacists] OR pharmacist*:ti,ab OR ("clinical" NEXT pharmacist*):ti,ab OR "community pharmacists":ti,ab OR [mh physicians] OR physician*:ti,ab OR "general practitioner":ti,ab OR doctor*:ti,ab) AND ([mh deprescriptions] OR deprescriptions:ti,ab OR deprescribing:ti,ab OR [mh "inappropriate prescribing"] OR "inappropriate prescribing":ti,ab OR De-prescribing:ti,ab OR ((medication*:ti,ab AND discontinu*:ti,ab) OR withdraw*:ti,ab)) AND ([mh "surveys and questionnaires"] OR questionnaire*:ti,ab OR survey*:ti,ab) AND ([mh "health knowledge, attitudes, practice"] OR Knowledge:ti,ab OR attitude*:ti,ab OR practice*:ti,ab OR [mh "Attitude of Health Personnel"]) | 460 |
| Scopus | 15/09/2024 | (INDEXTERMS(nurses) OR TITLE-ABS(nurse*) OR TITLE-ABS("registered nurses") OR INDEXTERMS(pharmacists) OR TITLE-ABS(pharmacist*) OR TITLE-ABS("clinical pharmacist*") OR TITLE-ABS("community pharmacists") OR INDEXTERMS(physicians) OR TITLE-ABS(physician*) OR TITLE-ABS("general practitioner") OR TITLE-ABS(doctor*)) AND (INDEXTERMS(deprescriptions) OR TITLE-ABS(deprescriptions) OR TITLE-ABS(deprescribing) OR INDEXTERMS("inappropriate prescribing") OR TITLE-ABS("inappropriate prescribing") OR TITLE-ABS(De-prescribing) OR ((TITLE-ABS(medication*) AND TITLE-ABS(discontinu*)) OR TITLE-ABS(withdraw*))) AND (INDEXTERMS("surveys and questionnaires") OR TITLE-ABS(questionnaire*) OR TITLE-ABS(survey*)) AND (INDEXTERMS("health knowledge, attitudes, practice") OR TITLE-ABS(Knowledge) OR TITLE-ABS(attitude*) OR TITLE-ABS(practice*) OR INDEXTERMS("Attitude of Health Personnel")) | 1692 |
| Web of Science | 13/09/2024 | (ALL=nurses OR (TI=nurse* OR AB=nurse*) OR (TI="registered nurses" OR AB="registered nurses") OR ALL=pharmacists OR (TI=pharmacist* OR AB=pharmacist*) OR (TI="clinical pharmacist*" OR AB="clinical pharmacist*") OR (TI="community pharmacists" OR AB="community pharmacists") OR ALL=physicians OR (TI=physician* OR AB=physician*) OR (TI="general practitioner" OR AB="general practitioner") OR (TI=doctor* OR AB=doctor*)) AND (ALL=deprescription OR (TI=deprescription OR AB=deprescription) OR (TI=deprescribing OR AB=deprescribing) OR ALL="inappropriate prescribing" OR (TI="inappropriate prescribing" OR AB="inappropriate prescribing") OR (TI=De-prescribing OR AB=De-prescribing) OR (((TI=medication* OR AB=medication*) AND (TI=discontinu* OR AB=discontinu*)) OR (TI=withdraw* OR AB=withdraw*))) AND (ALL="surveys and questionnaires" OR (TI=questionnaire* OR AB=questionnaire*) OR (TI=survey* OR AB=survey*)) AND (ALL="health knowledge, attitudes, practice" OR (TI=Knowledge OR AB=Knowledge) OR (TI=attitude* OR AB=attitude*) OR (TI=practice* OR AB=practice*) OR ALL="Attitude of Health Personnel") | 977 |
| Embase | 12/09/2024 | ('deprescription'/exp OR 'deprescriptions':ti,ab,kw OR 'deprescribing':ti,ab,kw OR 'prescribing error'/exp OR 'inappropriate prescribing':ti,ab,kw OR 'de-prescribing':ti,ab,kw OR (('medication*':ti,ab,kw AND 'discontinu*':ti,ab,kw) OR 'withdraw*':ti,ab,kw)) AND ('nurse'/exp OR 'nurse*':ti,ab,kw OR 'registered nurses':ti,ab,kw OR 'pharmacist'/exp OR 'pharmacist*':ti,ab,kw OR 'clinical pharmacist*':ti,ab,kw OR 'community pharmacists':ti,ab,kw OR 'physician'/exp OR 'physician*':ti,ab,kw OR 'general practitioner':ti,ab,kw OR 'doctor*':ti,ab,kw) AND ('surveys' AND 'questionnaire'/exp OR 'questionnaire*':ti,ab,kw OR 'survey*':ti,ab,kw) AND ('attitude to health'/exp OR 'knowledge':ti,ab,kw OR 'attitude*':ti,ab,kw OR 'practice*':ti,ab,kw OR 'health personnel attitude'/exp) | 2544 |
| CINAHL | 20/09/2024 | ((MH ( nurses or nursing staff or nurse) OR TI nurse* OR AB nurse* OR TI registered nurses OR AB registered nurses OR MH  (pharmacists or pharmacist) OR TI pharmacist* OR AB pharmacist* OR TI clinical pharmacist* OR AB clinical pharmacist* OR TI community pharmacist* OR AB community pharmacist*) OR (MH physicians OR TI physician* OR AB physician* OR TI general practitioner OR AB general practitioner OR TI doctor* OR AB doctor*)) AND ((MH deprescriptions OR TI deprescription* OR AB deprescription* OR TI (deprescribing or deprescribe or de-prescribe or de-prescribing) OR AB (deprescribing or deprescribe or de-prescribe or de-prescribing) OR TI inappropriate prescribing OR AB inappropriate prescribing OR TI de-prescribing OR AB de-prescribing OR TI medication discontinuation OR AB medication discontinuation)) AND ((MH (surveys and questionnaires) OR TI (surveys and questionnaires) OR AB (surveys and questionnaires) OR MH (surveys and questionnaires or quantitative) OR TI (surveys and questionnaires or quantitative) OR AB (surveys and questionnaires or quantitative) OR MH (questionnaire or survey or scale or instrument) OR TI (questionnaire or survey or scale or instrument) OR AB (questionnaire or survey or scale or instrument) OR MH (questionnaire or survey or scale or instrument or measurement or assessment) OR TI (questionnaire or survey or scale or instrument or measurement or assessment) OR AB (questionnaire or survey or scale or instrument or measurement or assessment)) AND ((MH health knowledge, attitudes, practice OR TI health knowledge, attitudes, practice OR AB health knowledge, attitudes, practice OR MH (health knowledge, attitudes, practice or knowledge, attitudes, practice) OR TI (health knowledge, attitudes, practice or knowledge, attitudes, practice) OR AB (health knowledge, attitudes, practice or knowledge, attitudes, practice) OR TI knowledge OR AB knowledge OR TI attitude* OR AB attitude* OR TI practice* OR AB practice*) OR (MH attitude of health personnel OR TI attitude of health personnel OR AB attitude of health personnel)) | 214 |
| Google Acadêmico | 15/09/2024 | (Nurses OR Pharmacists OR Doctors) AND (Deprescription OR Deprescribing) AND (Questionnaire OR Survey) AND (Knowledge OR Attitude OR Practice) | 200 |
| ProQuest | 13/09/2024 | TS=((Nurses OR Nurses OR Nurse OR "registered nurses" OR Pharmacists OR Pharmacists OR Pharmacist OR "clinical pharmacists" OR "clinical pharmacist" OR "community pharmacists" OR Physicians OR Physicians OR Physician OR "general practitioner" OR Doctor OR Doctors) AND (deprescription OR Deprescription OR Deprescribing OR "inappropriate prescribing" OR De-prescribing OR "medication discontinuation" OR "medication discontinuation") AND ("surveys and questionnaires" OR Questionnaires OR Questionnaire OR Surveys OR Survey) AND ("health knowledge, attitudes, practice" OR Knowledge OR Attitude OR Attitudes OR Practice OR Practices OR “Attitude of Health Personnel")) | 18 |
