# Supplementary material for: Instruments for assessing healthcare professionals’ knowledge, attitudes and practices regarding deprescribing: a scoping review
Source: Int J Clin Pharm. 2026 Aug 21;48(6):2201–11. doi: 10.1007/s11096-026-02215-y (PMC13731477; doi:10.1007/s11096-026-02215-y)
Supplement: Supplementary file 3 [file 11096_2026_2215_MOESM3_ESM.docx]

| **Data Extraction Form** | | |
| --- | --- | --- |
| **Part 1: Study Characteristics** | | |
| **Variable** | **Measure** | **Explanation** |
| Author | - | Write the last name of the first author of the article. |
| Year of publication | - | Write only the year of publication of the article (e.g., 2024). |
| Country of publication | Country where the study was conducted. | Select the country name from the dropdown list. If the country is not listed, select “Other”, and a field will open so that the country name can be entered.  Notes:  1) For studies conducted in England, select “United Kingdom”.  2) If the study was conducted in more than one country, select “Multinational”.  3) If the country where the study was conducted is not clearly stated in the article, select the country of the corresponding author. |
| Region* | East Asia & Pacific; Europe & Central Asia;  Latin America & Caribbean; Middle East & North Africa; North America; South Asia; Sub-Saharan Africa | East Asia & Pacific (Australia; China; Japan; Korea; New Zealand; Singapore; Philippines; Thailand)  Europe & Central Asia (Denmark; Finland; France; Germany; Hungary; Ireland; Netherlands; Poland; Portugal; Russian Federation; Spain; Sweden; United Kingdom)  Latin America & Caribbean (Argentina; Bolivia; Brazil; Chile; Colombia; Mexico; Panama; Paraguay; Puerto Rico; Uruguay)  Middle East & North Africa (Egypt; Saudi Arabia; Tunisia)  North America (Canada; United States)  South Asia (India)  Sub-Saharan Africa (South Africa)  If the country is not on this list, check the “World Bank 2023” spreadsheet, filter the “Country” column, and identify the region in the “Region” column. |
| Country income level* | Low-income; Lower-middle-income; Upper-middle-income; or High-income | Low-income:  Lower-middle-income (Bolivia; Egypt; India; Tunisia)  Upper-middle-income (Argentina; Brazil; China; Colombia; Mexico; Paraguay; South Africa; Thailand)  High-income (Australia; Canada; Chile; Denmark; Finland; France; Germany; Hungary; Ireland; Japan; Korea; Netherlands; New Zealand; Panama; Poland; Portugal; Puerto Rico; Russian Federation; Saudi Arabia; Singapore; Spain; Sweden; United Kingdom; United States; Uruguay)  If the country is not on this list, check the “World Bank 2023” spreadsheet, filter the “Country” column, and identify the country income classification in the “Income Group” column. |
| Study design | Quantitative studies; Validation studies; Mixed-methods studies; Other; Not reported | 1) Cross-sectional  2) Cohort  3) Case-control  4) Clinical trial  5) Validation studies  6) Mixed-methods studies: include only those in which knowledge, attitudes, and practices are analyzed quantitatively using an instrument.  7) Other: if none of the study designs listed above is clearly described in the article. Record the study description exactly as presented in the original text.  8) Not reported: if the study does not clearly indicate the study design, select “Not reported”.  Exclude: Qualitative studies; literature reviews (systematic, narrative, integrative); study protocols; commentaries, letters to the editor, and editorials; case reports and case series.  If an included article fits any of these categories, the article must be excluded and the reason must be recorded. |
| Deprescribing concept | Author-defined concept | Copy the deprescribing concept exactly as it is written in the article, in English or in the original language (this definition may appear either in the methods or in the introduction).  If the deprescribing concept appears in more than one section of the article, extract the concept defined in the methods section.  If there is no definition, write NA: Not applicable. |
| Study setting | Primary Care; Hospital; Long-Term Care Institution; Community Pharmacy; Outpatient Clinic / Specialized Clinic; Other; Not reported | Select all options that correspond to the content of the study; more than one option may be selected.  1) Primary Care (e.g., primary healthcare units, family health clinics, general practice offices).  2) Hospital (e.g., public or private hospitals, university hospitals).  3) Long-Term Care Institution (e.g., nursing homes, residential care facilities, geriatric institutions, sheltered housing).  4) Community Pharmacy (e.g., public or private pharmacies and drugstores open to the general public).  5) Outpatient Clinic / Specialized Clinic (e.g., public or private specialty outpatient clinics, private clinics).  6) Other: if none of the options listed above is described in the article, choose “Other”. In this case, a text box will be displayed so that you can write the study setting exactly as it appears in the article, in English or in the original language.  7) Not reported: when the article does not describe the study setting, select “Not reported”. |
| **Part 2: Population Characteristics** | | |
| **Variable** | **Measure** | **Explanation** |
| Participants | Nurse; Pharmacist; Physician | Select all options that correspond to the content of the study; more than one option may be selected.  When the study combines populations, such as “healthcare professionals + students”, “healthcare professionals + patients/caregivers/family members”, or “healthcare professionals (nurses, pharmacists, physicians) + other professionals (e.g., physiotherapists, nutritionists, dentists, etc.)”, exclude studies in which it is not possible to identify and extract data for nurses, pharmacists, and physicians separately.  If an included article fits any of the categories above, the article must be excluded and the reason must be recorded. |
| Total number of professionals included in the study | - | Record the absolute number.  If the data are provided only as relative frequencies, calculate the number in relation to the total.  If the study author does not provide the total number of participants, add up all professional categories. |
| Number of professionals by professional category | - | Record the absolute number.  If the data are provided only as relative frequencies, calculate the number in relation to the total.  If the study author does not provide the total number of participants, add up all professional categories. |
| **Part 3: Instrument Characteristics** | | |
| **Variable** | **Measure** | **Explanation** |
| Does the study present a questionnaire/instrument including the questions? | Yes/No | Does the study present a questionnaire/instrument including the questions?  1) Yes: The study includes the full questionnaire, or the questions are clearly described in the text, tables, appendices, or supplementary material. The questionnaire must be included in the methods, appendices, or supplementary material. The questions must be clearly described, together with their response options, allowing the structure of the instrument and the domains assessed to be identified.  2) No: The study only mentions that a “survey” or “questionnaire” was used, but does not present the questions or indicate where they can be accessed.  If the article does not provide the questionnaire content, even if it presents the results in tables with responses, the answer must be “No”, unless the content of the questions can be inferred unequivocally.  If the answer is “No”, stop the data extraction. |
| Tool | - | Record the name of the tool in the same language as the article. If the tool does not have a name, record NA (Not applicable). |
| Acronym | - | Record the acronym of the tool in the same language as the article. If the tool does not have an acronym, record NA (Not applicable). |
| Language | English; Spanish; Chinese (Mandarin); French; German; Portuguese; Russian; Arabic; Hindi; Japanese; Italian; Dutch; Swedish; Other; Multiple Languages; Not reported | 1) Select the language of the tool. In cases of validation or cross-cultural adaptation, record the language of the version validated in the study, not the original language of the tool.  Examples:  Case 1: The article reports a cross-cultural adaptation.  “The original version of the tool was developed in English, but this study validated the translated Spanish version.”  Correct entry: Spanish (because the validation in the article was conducted in that language).  Case 2: The study used the original English version of the tool.  “We used instrument X, originally published in English, without modifications.”  Correct entry: English.  2) Other: if none of the options listed above is described in the article, choose “Other”. In this case, a text box will be displayed so that you can write the language of the study.  3) Multiple Languages: if multiple languages are used (e.g., a study conducted in different countries), list all languages used.  4) Not reported: if the article does not explicitly report it. |
| Which domain was analyzed in the study?  Select all options that correspond to the content of the questionnaire; more than one option may be selected.  Not all questionnaires have the knowledge, attitudes, and practices domains clearly separated. In some cases, the questions may be mixed or may not have an explicit categorization.  To extract these data, analyze each question in the questionnaire/instrument according to the guidance provided and select the domain whenever at least one of the domains related to knowledge, attitudes, and practices is assessed.  If at least one question in the questionnaire addresses knowledge, attitude, or practice, the corresponding domain must be selected. If a domain is not represented in any question, the option “Other” must be selected.  Record the number of questions for each domain in the form (absolute number). | Knowledge | Operational definition: Understanding of deprescribing, including guidelines, criteria, and risks. (Does the study assess how much professionals know about deprescribing?)  Synonyms: Knowledge, Awareness, Understanding, Familiarity, Information, Education, Training, etc. |
|  | Attitudes | Operational definition: Professionals’ perceptions, beliefs, and willingness regarding deprescribing (e.g., confidence, predisposition). (Does the study assess how professionals perceive or feel about deprescribing?)  Synonyms: Attitude, Belief, Perception, Perspective, Opinion, Willingness, Confidence, Preference, etc. |
|  | Practices | Operational definition: What professionals actually do in relation to deprescribing (e.g., implementation, clinical decision-making). (Does the study measure professionals’ actual behavior or actions?)  Synonyms: Practice, Behavior, Action, Implementation, Intervention, Management, Strategy, etc. |
|  | Other | When the questionnaire/instrument does not assess any of the knowledge, attitudes, or practices domains, select “Other”. |
| Total number of domains | - | Domains are structured subdivisions within an assessment instrument, organized by specific themes or concepts. Each domain groups a set of questions that measure a distinct aspect of the phenomenon under study.  For example, a questionnaire on deprescribing may include domains such as ‘knowledge’, ‘attitudes’, and ‘practices’.  If the instrument does not explicitly present this division, it should be considered unidimensional and the number of domains should be recorded as ‘1’ (One). |
| Number of questions in the knowledge domain | - | Record the absolute number. |
| Number of questions in the attitudes domain | - | Record the absolute number. |
| Number of questions in the practices domain | - | Record the absolute number. |
| Number of questions of other types | - | Record the absolute number. |
| Total number of questions/statements/items | - | Record the absolute number. |
| Does the study present the response format used in the instrument questions? | Likert Scale; Visual Analog Scale; Dichotomous Scale; Ordinal Scale; Nominal/Categorical Scale; Other; Open-Ended Question; Not reported | If the instrument uses any scale*, proceed to the next question.  *A measurement scale is a structured set of items intended to quantify a concept through standardized responses, which may be categorical or continuous.  In our study, measurement scale refers to the response format used in the questionnaire questions. To answer this question, you should examine the response options for each question. For example, responses such as “Yes/No”, “Agree/Disagree”, “1 to 5”, or multiple choice without hierarchy are all types of response scales.  Select all options that correspond to the content of the study; more than one option may be selected.  1) Likert Scale (e.g., 3, 4, 5, 7 points)  2) Visual Analog Scale (VAS) (e.g., continuous line from 0 to 10)  3) Dichotomous Scale (e.g., Yes/No, True/False)  4) Ordinal Scale (e.g., Low/Medium/High, Poor/Fair/Good)  5) Nominal/Categorical Scale (e.g., multiple choice without hierarchy)  6) Other (Specify)  7) Open-Ended Question  8) Not reported |
| Does the instrument generate a quantitative score? | Sum of items; Mean of responses; Differential weighting by item; Other; Not reported | If the instrument generates any score*, proceed to the next question.  *The score of a measurement instrument refers to the numerical value resulting from the application of a set of items or questions, representing a specific construct or attribute. It may be derived through different methods, such as summing responses, weighted averages, or advanced statistical models.  Select all options that correspond to the content of the study; more than one option may be selected.  1) Sum of items (sum of the scores of all questions)  2) Mean of responses (average of participants’ response values)  3) Differential weighting by item (some questions carry more weight than others)  4) Other (Specify) (text field for description)  5) Not reported |
| Minimum score value | - | Record the absolute number.  If there is no value, record NI: Not informed. |
| Maximum score value | - | Record the absolute number.  If there is no value, record NA: Not applicable. |
| Does the study present validation results for the questionnaire? | Yes/No | If the study presents validation results*, proceed to the next question. The study must present validation metrics.  *Validation of an instrument refers to the process of evaluating its ability to measure the intended concept accurately and consistently. This process may include different types of validity (content, criterion, and construct) and reliability, ensuring that the instrument is appropriate for its proposed use.  If the study only reports that the questionnaire used is validated, and even presents the validity measures of the original instrument, but does not present any validity metric in the study being analyzed, select “No”.  Select all options that correspond to the content of the study; more than one option may be selected. |
| Internal Consistency | Yes/No | Description: Assesses the correlation among items within the same instrument domain.  Statistics: Cronbach’s alpha, McDonald’s Omega |
| Reliability: Test-Retest | Yes/No | Description: Measures the stability of the instrument over time.  Statistics: Intraclass Correlation Coefficient (ICC), Pearson, Spearman |
| Reliability: Inter-Rater | Yes/No | Description: Measures agreement between different raters applying the same instrument.  Statistics: Cohen’s Kappa Coefficient, Intraclass Correlation Coefficient (ICC), Percentage Agreement |
| Reliability: Intra-Rater | Yes/No | Description: Measures the stability of responses when the same rater applies the instrument at different time points.  Statistics: Intraclass Correlation Coefficient (ICC), Bland-Altman, Agreement Test |
| Measurement Error: Test-Retest | Yes/No | Description: Measures variation across repeated administrations.  Statistics: Standard Error of Measurement (SEM), Coefficient of Variation (CV), Bland-Altman |
| Measurement Error: Inter-Rater | Yes/No | Description: Measures variation in results between raters.  Statistics: Standard Error of Measurement (SEM), Difference Between Raters |
| Measurement Error: Intra-Rater | Yes/No | Description: Measures variation in the responses of the same rater.  Statistics: Standard Error of Measurement (SEM), Bland-Altman |
| Content Validity | Yes/No | Description: Assesses whether the instrument measures all relevant aspects of the intended concept.  Statistics: Content Validity Index (CVI) |
| Criterion Validity: Concurrent | Yes/No | Description: Measures the correlation with another validated instrument applied simultaneously.  Statistics: Pearson/Spearman Correlation, Linear Regression |
| Criterion Validity: Predictive | Yes/No | Description: Measures the ability of the instrument to predict a future outcome.  Statistics: Logistic Regression, ROC Curve (AUC) |
| Construct Validity: Structural | Yes/No | Description: Measures the factor structure of the instrument.  Statistics: Exploratory Factor Analysis (EFA), Confirmatory Factor Analysis (CFA), Structural Equation Modeling (SEM) |
| Construct Validity: Hypothesis Testing | Yes/No | Description: Measures whether the instrument correctly differentiates expected subgroups.  Statistics: Group Comparison (t-test, ANOVA, Chi-square), Pearson/Spearman Correlation |
| Construct Validity: Cross-Cultural | Yes/No | Description: Measures whether a translated instrument maintains conceptual equivalence with the original version.  Statistics: Descriptive statistics |
| *https://www.worldbank.org | |  |
