# Supplementary material for: Instruments for assessing healthcare professionals’ knowledge, attitudes and practices regarding deprescribing: a scoping review
Source: Int J Clin Pharm. 2026 Aug 21;48(6):2201–11. doi: 10.1007/s11096-026-02215-y (PMC13731477; doi:10.1007/s11096-026-02215-y)
Supplement: Supplementary file 4 [file 11096_2026_2215_MOESM4_ESM.docx]

| **Supplementary Table 4: Knowledge, attitudes and practices domain combinations assessed across included instruments (n=40)** | **n (%)** |
| --- | --- |
| Knowledge + Attitudes + Practices | 12 (30.0%) |
| Attitudes + Practices | 11 (27.5%) |
| Attitudes | 10 (25.0%) |
| Practices | 3 (7.5%) |
| Knowledge + Attitudes | 2 (5.0%) |
| Knowledge + Practices | 1 (2.5%) |
| Knowledge alone | 0 (0.0%) |
| Other | 1 (2.5%) |
| **Total** | **40 (100%)** |
