# Supplementary material for: Instruments for assessing healthcare professionals’ knowledge, attitudes and practices regarding deprescribing: a scoping review
Source: Int J Clin Pharm. 2026 Aug 21;48(6):2201–11. doi: 10.1007/s11096-026-02215-y (PMC13731477; doi:10.1007/s11096-026-02215-y)
Supplement: Supplementary file 5 [file 11096_2026_2215_MOESM5_ESM.docx]

| **Supplementary Table 5: Characteristics and key findings of included studies with reported Knowledge, Attitudes and Practices assessment instruments** | | | | | | | | | |
| --- | --- | --- | --- | --- | --- | --- | --- | --- | --- |
| **Author** | **Year** | **Country** | **Region** | **Income** | **Study Type** | **Study Setting** | **Healthcare Professional** | **Domain** | **Instrument** |
| Chia | 2018 | Singapore | East Asia & Pacific | High income | Cross-sectional | Primary Care | Nurse Pharmacist Physician | Other | Not Validated |
| Saka | 2024 | Nigeria | Sub-Saharan Africa | Low-income | Cross-sectional | Outpatient Clinic/Hospital | Nurse Pharmacist Physician | Attitudes Practices  Other | Validated |
| Tegegn | 2021 | Ethiopia | Sub-Saharan Africa | Low-income | Cross-sectional | Hospital | Nurse Pharmacist Physician | Attitudes  Other | Not Validated |
| Rossi | 2020 | Italy | Europe & Central Asia | High income | Cross-sectional | Primary Care | Physician | Attitudes | Not Validated |
| Nadarajan | 2018 | Singapore | East Asia & Pacific | High income | Cross-sectional | Hospital | Physician | Knowledge Attitudes Practices | Not Validated |
| Agosti | 2024 | Italy | Europe & Central Asia | High income | Validation | Outpatient Clinic/Specialized Clinic/Hospital | Physician | Knowledge Attitudes Practices  Other | Validated |
| Jawahar | 2023 | India | South Asia | Low-middle-income | Cross-sectional | Hospital | Physician | Knowledge Attitudes Practices | Not Validated |
| White | 2019 | Australia | East Asia & Pacific | High income | Cross-sectional | Primary Care | Physician | Attitudes | Validated |
| Gillespie | 2019 | Australia | East Asia & Pacific | High income | Cross-sectional | Primary Care | Physician | Knowledge Attitudes Practices | Not Validated |
| Abu-Fahra | 2024 | Jordan | Middle East & North Africa | Low-middle-income | Cross-sectional | Primary Care/Hospital | Physician | Knowledge Attitudes Practices | Validated |
| Aggarwal | 2022 | India | South Asia | Low-middle-income | Cross-sectional | Hospital | Physician | Knowledge Attitudes Practices | Not Validated |
| Davila | 2021 | United States | North America | High income | Cross-sectional | Primary Care | Nurse Pharmacist Physician | Attitudes Practices Other | Not Validated |
| Llor | 2022 | Spain | Europe & Central Asia | High income | Cross-sectional | Primary Care | Physician | Attitudes Practices | Not Validated |
| Gray | 2022 | United States | North America | High income | Cross-sectional | Primary Care | Physician | Knowledge Attitudes Other | Not Validated |
| Akande-Sholabi | 2023 | Nigeria | Sub-Saharan Africa | Low-middle-income | Cross-sectional | Hospital | Physician | Knowledge Attitudes Practices | Validated |
| Sweta | 2019 | India | South Asia | Low-middle-income | Cross-sectional | Not Reported | Physician | Attitudes | Not Validated |
| Bilbao | 2020 | Spain | Europe & Central Asia | High income | Cross-sectional | Primary Care | Physician | Attitudes | Not Validated |
| Kujiper | 2022 | Netherlands | East Asia & Pacific | High income | Cross-sectional | Outpatient Clinic/Specialized Clinic | Nurse Physician | Attitudes Practices | Not Validated |
| Mejias-Trueba | 2023 | Spain | Europe & Central Asia | High income | Cross-sectional | Primary Care/Hospital | Physician | Attitudes Practices Other | Not Validated |
| Niznik | 2022 | United States | North America | High income | Mixed-methods | Primary Care | Nurse Physician | Knowledge Attitudes Practices | Validated |
| Huffmyer | 2021 | United States | North America | High income | Cross-sectional | Primary Care/Community Pharmacy | Pharmacist Physician | Attitudes Practices | Not Validated |
| Amedi | 2024 | Norway | Europe & Central Asia | High income | Cross-sectional | Community Pharmacy | Pharmacist | Knowledge Attitudes Practices  Other | Not Validated |
| Peretti | 2018 | Canada | North America | High income | Mixed-methods | Long-Term Care Facility | Physician | Practices | Not Validated |
| Silva | 2022 | Portugal | Europe & Central Asia | High income | Cross-sectional | Not Reported | Physician | Practices | Not Validated |
| Lopes | 2021 | Portugal | Europe & Central Asia | High income | Cross-sectional | Primary Care | Physician | Practices | Validated |
| El-Dahiyat | 2023 | United Arab Emirates | Middle East & North Africa | High income | Cross-sectional | Community Pharmacy | Pharmacist | Knowledge Practices | Validated |
| Heinrich | 2021 | Ireland | Europe & Central Asia | High income | Cross-sectional | Community Pharmacy | Pharmacist | Knowledge Attitudes | Not Validated |
| Shresta | 2022 | Australia | East Asia & Pacific | High income | Validation | Primary Care/Hospital | Nurse Pharmacist Physician | Attitudes | Validated |
| Coelho | 2024 | Portugal | Europe & Central Asia | High income | Cross-sectional | Primary Care | Physician | Attitudes Practices Other | Not Validated |
| Djatche | 2018 | Italy | Europe & Central Asia | High income | Cross-sectional | Primary Care | Physician | Attitudes | Not Validated |
| Jungo | 2021 | Multinational | - | - | Cross-sectional | Not Reported | Physician | Attitudes Practices Other | Not Validated |
| Goyal | 2020 | United States | North America | High income | Cross-sectional | Outpatient Clinic/Specialized Clinic | Physician | Attitudes Practices | Not Validated |
| Mantelli | 2018 | Switzerland | Europe & Central Asia | High income | Cross-sectional | Primary Care | Physician | Attitudes Practices Other | Not Validated |
| Farrell | 2018 | Canada | North America | High income | Cross-sectional | Long-Term Care Facility/Primary Care | Nurse Pharmacist Physician | Attitudes | Validated |
| Bhat | 2018 | India | South Asia | Low-middle-income | Validation | Not Reported | Physician | Attitudes | Validated |
| Chróinín | 2015 | Multinational | - | - | Cross-sectional | Not Reported | Physician | Attitudes | Not Validated |
| Poelgeest | 2022 | Multinational | - | - | Cross-sectional | Primary Care/Long-Term Care Facility/Primary Care | Physician | Knowledge Attitudes Practices | Not Validated |
| Buzancic | 2023 | Croatia | Europe & Central Asia | High income | Cross-sectional | Primary Care/Community Pharmacy | Pharmacist Physician | Knowledge Attitudes Practices | Not Validated |
| Norton | 2023 | United States | North America | High income | Cross-sectional | Primary Care | Physician | Attitudes Practices | Validated |
| Buzancic | 2022 | Croatia | Europe & Central Asia | High income | Cross-sectional | Primary Care/Community Pharmacy | Pharmacist Physician | Knowledge Attitudes Practices | Validated |
